# Exploring the Role of Medicinal Plant-Based Immunomodulators for Effective Therapy of Leishmaniasis

**DOI:** 10.3389/fimmu.2014.00193

**Published:** 2014-05-05

**Authors:** Garima Chouhan, Mohammad Islamuddin, Dinkar Sahal, Farhat Afrin

**Affiliations:** ^1^Parasite Immunology Laboratory, Department of Biotechnology, Jamia Hamdard (Hamdard University), New Delhi, India; ^2^Malaria Group, International Centre for Genetic Engineering and Biotechnology, New Delhi, India

**Keywords:** medicinal plants, immunomodulators, leishmaniasis, antileishmanial, phytochemicals

## Abstract

Leishmaniasis is a pestilent affliction that importunately needs better therapeutics necessitated by the absence of effective vaccine, emergence as HIV co-infection, and the dread of debilitating chemotherapy. The *Leishmania* parasites incapacitate host macrophages by preventing the formation of phagolysosomes, impeding antigen presentation to T cells, leading to suppression of cell-mediated immunity. An ideal approach to cure leishmaniasis includes administration of antileishmanial compounds that can concomitantly establish an effective Th1 response via restoration of requisite signaling between macrophages and T cells, for subsequent activation of macrophages to eliminate intracellular amastigotes. Plants have provided an opulent treasure of biomolecules that have fueled the discovery of antileishmanial drugs. Modulation of immune functions using medicinal plants and their products has emerged as an effective therapeutic strategy. Herein, we review the plant extracts and natural products that have resulted in therapeutic polarization of host immunity to cure leishmaniasis. These immunostimulatory phytochemicals as source of potential antileishmanials may provide new strategies to combat leishmaniasis, alone or as adjunct modality.

## Introduction

Leishmaniasis is a neglected; usually poverty-associated complex vector-borne disease that is caused by more than 21 species of parasites belonging to the order Kinetoplastida and family Trypanosomatidae. The disease manifests into different clinical indexes depending on the parasite tropism and ranges from self-healing cutaneous lesions, to malign mucocutaneous leishmaniasis and fatal visceral manifestations. The disease is endemic in 98 countries, where 10–12 million people are afflicted worldwide with 1.5–2.5 million new cases, death toll of 70,000 and 350 million at risk of developing the infection (1–3). Since *Leishmania* parasites reside and multiply within the parasitophorous vacuoles of macrophages, failure of host immunity to contain the infection results in immunosuppression. Thus, the host becomes susceptible to various secondary infections including HIV (4, 5).

The antiquated therapeutic modalities for leishmaniasis are crippled because of variable efficacy, drug resistance, and pronounced side effects. Even the known antileishmanial drugs such as amphotericin B (AmB), sodium stibogluconate (SSG), and miltefosine exert their antileishmanial effect via host immunomodulation [(6) and references therein]. Modulation of the host immune response via generation of antileishmanial vaccine would certainly be a propitious step in leishmaniasis control, but is impeded by the digenetic life cycle of *Leishmania*, and antigenic diversity among different *Leishmania* species, making prospects of a cross-protective vaccine a distant future (7, 8). Thus, in the absence of any vaccine, a quintessential approach to control leishmaniasis shall be based on discovery of drugs from alternative sources that can directly kill the parasite as well as activate sentinels of immune system for clearance of the pathogen. Herein, we further elaborate the mechanisms employed by *Leishmania* parasites to evade host immune responses, lacunae in current chemotherapy and discuss potential role of natural immunomodulators in antileishmanial therapy.

## *Leishmania* Parasite-Evasion from Host Immune Defenses

### Modulation of neutrophil functions

Within 24 h of *Leishmania* infection, neutrophils are recruited to the site of infection, serving as early and transitory host to *Leishmania* promastigotes (9). Although exact mechanisms underlying the recruitment of neutrophils remain unclear, the role of both parasites or vector-derived molecules is speculated (9, 10). The role of neutrophils in *Leishmania* infection is variable, *Leishmania* species specific, and also depends on the host genetics (11–14). Although neutrophils are responsible for early containment of different *Leishmania* species (15–18), they play equally pivotal role in harboring the parasites till they reach their evolutionary destined host cells, i.e., macrophages. *Leishmania* promastigotes deviously modulate neutrophil phagocytic functions in more than one way. Internalized *Leishmania* promastigotes block the production of CXC-chemokine interferon gamma (IFN-γ) inducible protein-10, which results in decreased recruitment and activation of natural killer (NK) cells and Th1 cells (10). *Leishmania donovani* promastigotes have been shown to induce NETs (fibrous traps of DNA, histones, and proteins) in which they get trapped but escape their microbicidal activity by aid of lipophosphogylcan (LPG) (19). *Leishmania* parasites also extend the life span of neutrophils and delay their apoptosis by various mechanisms (20, 21). Since ingestion of apoptotic neutrophils by macrophages does not trigger macrophage microbicidal defenses, it creates safe passage for stealth entry of *Leishmania* parasites. The theory that neutrophils act as Trojan horses is well perceived in case of *L. donovani* (10, 22), but there is also evidence that *Leishmania* parasites escape neutrophils before infecting the macrophages (23), a Trojan rabbit strategy where viable promastigotes hide in the shadow of apoptotic neutrophils (9).

### Entry into macrophages

In their quest for survival, *Leishmania* parasites face the arduous challenge to gain entry inside the macrophages and silence their impeccable defenses. The parasites express a wide array of ligands on their surfaces, which interact with a variety of macrophage receptors. Some of the key receptors that mediate promastigote–macrophage binding include the receptors for complement, fibronectin, mannose–fucose, and other sugars [(24) and references therein]. Interestingly, the foremost ligands employed by parasite for its phagocytic uptake are not encoded by the parasite itself; instead, parasite gets ingested into the macrophages via opsonin-dependent pathways. The sharp-witted *Leishmania* parasites not only circumvent complement-mediated lysis but also modulate the complement system for their active uptake inside the macrophages. C3bi and C3b are two major complement system components that bind to promastigote surface (25–28) and facilitate their intracellular uptake via CR3 and CR1, respectively. The uptake via CR3 is more advantageous since internalization via these receptors does not result in oxidative burst and also suppresses the secretion of IL-12 and other pro-inflammatory signals, thus hampering the initiation of cell-mediated immunity (CMI) (29, 30).

### Establishing infection in macrophages

Once inside the macrophage phagosomes, promastigotes create an intracellular niche for their survival by silencing the macrophages through multifarious schemes. Predominantly, *Leishmania* parasites retard phagosome maturation, delay enodosome–phagosome fusion, inhibit hydrolytic enzymes in phagolysosomes, prevent generation of reactive-nitrogen and -oxygen species, suppress antigen presentation, and repress pro-inflammatory cytokine production. LPG present on the surface of *Leishmania* parasites retards phagosome maturation by inducing Cdc42- and Rac1 (Rho family GTPases, F-actin regulators)-dependent F-actin accumulation, which also involves inhibition of PKCα leading to impaired recruitment of LAMP-1 and rab7 (31–34). It has been reported earlier that incorporation of LPG selectively into one of the leaflets of lipid bilayers of phagosome membrane alters its biophysical properties making it less fusogenic [(31) and references therein]. LPG impairs the acquisition of vesicular proton-ATPase, which is involved in acidification of phagolysosomes (35). *Leishmania* parasites either decimate or suppress expression of major histocompatibility complex (36–38). *Leishmania* parasites also interfere with protein expression of their host cells (39), suppress the secretion of pro-inflammatory cytokines (IFN-γ, TNF-α, IL-6, IL-12), and induce secretion of anti-inflammatory cytokines (IL-4, IL-10, and TGF-β) via modulation of host cell signaling (40–42). Thus, establishment of *Leishmania* infection involves complex in-depth interactions between a vast repertoire of immunostimulating and immunosuppressive molecules that finally determine a species-dependent outcome of infection.

## Therapeutic Modalities

The pentavalent antimonials, SSG and Meglumine Antimoniate (MA), have been employed in treatment of visceral leishmaniasis (VL) and cutaneous leishmaniasis (CL) for more than 60 years. The use of SSG and MA as first-line treatment for VL has been already abandoned after failure rates of ~65% in endemic regions of Bihar, India. Antimonials show variable efficacy against CL and VL, and accompanying severe side effects have demerited their use. The second line of drug AmB is now employed to treat the antimony-resistant patients but the need of hospitalization, prolonged duration of treatment, and infusion-related side effects are drawbacks. These constraints have now been overcome by lipid formulations of AmB that prevent tissue retention, thereby reducing the toxicity and promoting preferential uptake by reticuloendothelial cells that harbor the parasites. However, cost of liposomal AmB is a serious limitation and frequent noxious effects and drug resistance associated with pentamidine has also led to its withdrawal. Paromomycin has been registered for use in India against VL but is oto- and nephrotoxic. Miltefosine, the first oral drug, is of limited use because of its teratogenicity (43–45).

## Natural Immunomodulators: Role in Antileishmanial Therapy

The drug discovery against leishmaniasis has been more reliant on therapeutic switching rather than discovery of novel drugs. Since elevation of host immunity is critical in parallel to the drug-mediated killing of *Leishmania* parasites, the antileishmanial arsenal may be benefited by antileishmanials that can prong a bifurcated attack, i.e., elimination of the parasites as well as restoration of CMI. The potential of immunomodulators in treating experimental leishmaniasis gained momentum with the discovery of antileishmanial activity of imiquimod (46, 47), an agonist for toll-like receptor 7, which is present on macrophages and dendritic cells (DC) and promotes the development of Th1 immune response [(48) and references therein]. Several other synthetic compounds such as S_2_ complex (an organic complex of copper chloride, ascorbic acid, and nicotinamide) (49), acetyl salicylic acid (50), and immunomodulatory peptide from cystatin (51) have been demonstrated to possess dual immune-modulating and antileishmanial activities.

Various herbal formulations and plant secondary metabolites such as flavonoids, isoflavonoids, saponins, alkaloids, sesquiterpenes, polysaccharides, tannins, indoles, and glucans are known to be immunomodulatory in different diseases (52, 53). Among natural resources, plants have been most extensively explored for bioactive leishmanicidal and immunomodulatory compounds. Plant extracts contain a plethora of biomolecules that can naturally kill *Leishmania* parasites and also exert immunostimulatory properties, on otherwise depressed immune system during the diseased state, as has been extensively reviewed in Table 1.

**Table 1:** **Immunomodulatory antileishmanial plant extracts or purified molecules thereof**.

Plant extracts/purified compounds/secondary metabolites/herbal medicines	*Leishmania* strain	Concentration (*in vitro*)/dose regimen (*in vivo*)	Immunomodulatory mechanism	Reference
*Asparagus racemosus* (whole plant)	*L. donovani* (Dd8)	^b^650 mg/kg b.w. +cisplatin (5 mg/kg b.w.) for 5 days i.p.	↑ INF-γ, IL-2, IgG2a	Sachdeva et al. (54)
			↓ IL-4, IL-10, IgG1	
			Induced DTH response	
*Allium sativum* in combination with *Tridax procumbens*	*Leishmania major* [Hd-18-(MHET/MX/97/Hd-18)]	^b^40 mg/kg b.w. (1:1) i.p. daily for 2 weeks	↑ IgG2a/IgG1 ratio	Gamboa-Leon et al. (55)
Galactomannan (isolated from seeds of *Mimosa scabrella*)	*Leishmania amazonensis* (MHOM/BR173/M2269)	250 μg/ml	↑ IL-1β, IL-6 and NO production	Adriazola et al. (56)
			TNF-α and IL-10 levels unaffected	
^a^Licarin A (neolignan, plant secondary metabolite)	*L. major* (MHOM/IL/1980/FN)	5 and 20 μg/ml	↓ IL-6 and IL-10	Néris et al. (57)
			No significant alterations in TNF-α and NO levels	
Niranthrin (lignan isolated from aerial parts of *Phyllanthus amarus*)	*L. donovani* (MHOM/IN/1983/AG83)	^b^5 and 10 mg/kg b.w. twice for 3 weeks	↑ NO, ROS, iNOS	Chowdhury et al. (58)
			Induced lymphoproliferation	
			↑ IFN-γ, TNF-α, and IL-12p70	
			↑ IgG2a levels	
			↓ IL-10 and TGF-β	
			No change in IL-4 expression and IgG1	
^a^Berberine chloride (quaternary isoquinoline alkaloid)	*L. donovani* isolate (NS2)	2.5 and 10 μM	↑ NO production	Saha et al. (59)
			Activated iNOS	
			↑ mRNA expression of IL-12p40	
			↓ IL-10	
			Upregulated p38 MAPK pathway	
Picroliv (iridoid glycoside mixture from *Picrorhiza kurroa*) in combination with fluconazole and miltefosine	*L. donovani* (MHOM/IN/80/Dd8)	Picroliv (10 mg/kg) + Fluconazole (50 mg/kg) + Miltefosine (5 mg/kg b.w.) in hamsters	Induced lymphoproliferation	Shakya et al. (60)
			↑ ROS, hydrogen peroxide, RNS	
			↑ Phagocytic activity	
*Spiranthera odoratissima* (fruit hexane extract and its alkaloid Skimmianine)	*Leishmania braziliensis*	1.6 μg/ml	↑ NO production	Dos Santos et al. (61)
			↓ IL-10 production	
*Echium amoenum* (flowers-aqueous and alcoholic extracts)	*L. major* (MRHO/IR/75/ER)	^b^250, 750, and 3750 mg/kg b.w.	↑ IFN-γ	Hosseini and Abolhassani (62)
			Induced lymphoproliferation	
			IL-4 levels unaffected	
*A. sativum* (aqueous extract)	*L. major* (MRHO/IR/75/ER)	37 mg/ml	↑ INF-γ and iNOS mRNA expression levels	Gharavi et al. (63)
*A.sativum* (aqueous extract)	*L. major* (MRHO/IR/75/ER)	37 mg/ml	↑ IL-12	Gharavi et al. (64)
			↓ IL-10	
^a^Artemisinin (sesquiterpene lactone from *Artemisia annua*)	*L. donovani*	10 and 25 μM	↑ NO production	Sen et al. (65)
		^b^10 and 25 mg/kg b.w.	↑ IL-12 and IFN-γ	
*Kalanchoe pinnata* (leaves-aqueous extract)	*Leishmania chagasi*	^b^400 mg/kg b.w. by intragastric gavage from day 1–29 of infection	Depressed serum IgG levels	Gomes et al. (66)
			↓ IL-4, INF-γ	
			↑ NO production	
*Warburgia ugandensis, Psiadia punctulata*, and *Chasmanthera dependens* (bark-aqueous extract)	*L. major* (IDU/KE/83 = NLB-144)	1000 μg/ml	↑ NO production	Githinji et al. (67)
*Himatanthus sucuuba* latex	*L. amazonensis* (WHOM/BR/75/Josefa strain)	200 μg/ml	↑ TNF-α and NO production	Soares et al. (68)
			↓ TGF-β	
*A.sativum* (methanolic extract)	*L. donovani* (NLB065)	250 μg/ml	↑ NO production	Wabwoba et al. (69)
*Xylopia discreta* (leaf methanolic extract and essential oil)	*L. panamensis* (MHOM/CO/87/UA140)	Different concentrations	↑ Monocyte chemoattractant protein-1 (MCP-1) expression	López et al. (70)
^a^Quassin (one of the quassinoids isolated from *Quassia amara*)	*L. donovani* (MHOM/IN/1983/AG83)	25 μg/ml	↑ iNOS2 expression	Bhattacharjee et al. (71)
			↑ TNF-α, and IL-12p70	
			↓ TGF-β and IL-10	
*A.sativum* (aqueous extract)	*Leishmania mexicana* (MNYC/BZ/62/M379)	37 μg/ml	↑ NO production	Gamboa-León et al. (72)
		^b^20 and 60 mg/kg b.w. for 2 weeks i.p.	↑ IFN-γ	
*Pelargonium sidoides* (aqueous-ethanolic formulation of roots and methanol insoluble fraction of this extract)	*L. major*	50 μg/ml	↑ iNOS activity ↑ IFN-γ, IL-12, IL-18 mRNA levels	Trun et al. (73)
^a^Plant polyphenols (Tannins and structurally related compounds)	*L. major* and *L. donovani* promastigotes	Different concentrations	Moderate effect on NO production ↑ TNF and INF like activities	Kolodziej and Kiderlen (74)
*Desmodium gangeticum* (Aminoglucosyl glycerolipid and Cerebroside)	*L. donovani*	100 μg/ml	↑ NO production	Mishra et al. (75)
Canova medication (*Aconitum napellus, Arsenium album, Bryonia alba*, and *Thuya occidentalis)*	*L. amazonensis* (MHOM/BR/73/M2269)	20 and 40%	↑ NO production	Pereira et al. (76)
*Croton cajucara* (Essential oil)	*L. amazonensis* (Raimundo strain, MHOM/BR/76/Ma-5)	1, 1.5, and 0.2 ng/ml	↑ NO production	do Socorro et al. (77)
*A.sativum* extract	*L. major* (MRHO/IR/76/ER)	^b^Garlic (20 mg/kg b.w.) +glucantime (60 mg/kg b.w.) daily for 2 weeks	↑ IFN-γ and IL-2 ↓IL-4 and IL-10	Ghazanfari et al. (78)

*^a^ Synthetic molecules of plant origin*.

*^b^ Studies carried out in BALB/c mice*.

In brief, the studies examining immunomodulatory effect of bioactive plant extracts or compounds have reported skewing of immune response from Th2 (diseased state) to Th1 (cure) by causing the up- or downregulation of pro-inflammatory (activating Th1) and anti-inflammatory (promoting Th2) cytokines, respectively. The most commonly assessed immunomodulatory parameter for parasite clearance is stimulation of nitric oxide (NO). NO is the principle effector molecule in killing of *Leishmania* amastigotes (79) and is either estimated directly as nitrite concentration in culture supernatant or indirectly by the changes in nitric oxide synthase (iNOS) gene expression levels. NO-mediated killing of *Leishmania* parasites by tannins and related compounds has also been demonstrated (74). IL-12 is the central cytokine produced by DC, NK, and T cells, which activates macrophages to produce IFN-γ and TNF-α. Different plant secondary metabolites and extracts have induced IL-12 up-regulation (Table 1), indicating the worthy potential of natural resources. Macrophages also produce IL-18, which in synergism with IL-12 stimulates IFN-γ production and aids in parasite clearance (80, 81). *Pelargonium sidoides* extracts have been shown to increase mRNA levels of IL-18 in *Leishmania major* infection (73).

In parallel to stimulation of IL-12 and other pro-inflammatory cytokines, expression levels of IL-4, IL-10, and TGF-β have also been widely investigated (Table 1). IL-4, IL-10, and TGF-β inhibit the production of IFN-γ from macrophages. IL-4 is known to potently inhibit macrophage activation, but IL-10 plays a cardinal role in progression of both CL and VL. In both, murine and human VL, despite the production of adequate amounts of IFN-γ, the hosts are unable to mount an effective CMI response, and this host inefficiency is attributed to increased levels of IL-10. Kane and Mosser (82) demonstrated that host-derived IgG present on *Leishmania* amastigotes ligates to Fcγ receptors on inflammatory macrophages and modulates them to secrete IL-10 in high amounts in CL. The levels of these Th2 cytokines have been observed to decline along with successful recuperation of CMI after treatment with immunomodulatory extracts and molecules of natural origin (Table 1).

It can thus be well established that natural immunomodulators can skew the Th1–Th2 balance but pro- and anti-inflammatory cytokines play diverse and inter-regulatory roles. As also supported by Couper et al. (83), understanding the dynamics of Th1–Th2 paradigm has changed over the years and it is conceived that both Th1 and Th2 cells can mediate inflammation as well as aid parasite clearance. For instance, Néris et al. (57) reported that Licarin A, treated *L. major*-infected macrophages exhibit decline in IL-6 as well as IL-10 levels. IL-6 is a characteristic pro-inflammatory cytokine, which also negatively regulates Th1 differentiation and promotes CD4^+^ Th2 differentiation mediated by IL-4 (84). However, as the study presented only the *in vitro* data, and also the pro- or anti-immunopotentiating effect can be dose dependent (85) further *in vivo* studies may throw proper light on mechanism of action of Licarin A.

## Conclusion

The use of herbal preparations to modulate the immune response to cure or avert diseases has been described in traditional systems of Indian, Unani, and Chinese medicine. The natural substances with dual, antileishmanial, and immunomodulatory properties have been carefully evaluated, however, it should be noted that human leishmaniasis varies in immunological pattern from murine leishmaniasis. Thus, the natural immunomodulators need to be evaluated more systematically and specifically in terms of dosage, biodistribution, kinetics, and interactions with other drugs and their putative role in other co-infections should also be examined.

Nonetheless, the concept of using natural immunomodulators to treat parasitic infections including leishmaniasis holds mighty potential in achieving the control of this disease. These natural immunomodulatory molecules can serve as scaffolds for synthesis and discovery of new immunodrugs. Also, the use of natural immunomodulators in synergy with existing drugs may involve the functional manipulation of multiple molecular targets leading to improved therapeutic efficacy and reduced toxicity.

## Conflict of Interest Statement

The authors declare that the research was conducted in the absence of any commercial or financial relationships that could be construed as a potential conflict of interest.
